# Interleukin-35 inhibited the production of histamine and pro-inflammatory cytokines through suppression MAPKs pathway in HMC-1 cells

**DOI:** 10.1186/s13223-021-00543-4

**Published:** 2021-04-17

**Authors:** Li-xin Fu, Tao Chen, Qiao-mei Sun, Pei-mei Zhou, Zai-pei Guo

**Affiliations:** 1 Department of Dermatovenereology, Chengdu Second People’s Hospital, Chengdu, 610017 Sichuan China; 2 Department of Dermatovenereology, Chengdu Qingbaijiang Distinct People’s Hospital, Chengdu, 610300 Sichuan China; 3 Department of Dermatovenereology, West China Hospital of Sichuan University, No. 37, Guoxue Alley, Chengdu, 610041 Sichuan China

**Keywords:** Interleukin-(IL)35, Mast cells, Histamine, Anti-inflammatory effect, MAPK

## Abstract

**Background:**

IL-35 is a newly anti-inflammatory cytokine that belongs to the IL-12 family. Mast cells, as one of the major effector cells in the immune response system, plays an important role in the pathogenesis of chronic spontaneous urticarial (CSU). Our study aims to explore the inhibited role of IL-35 in HMC-1.

**Methods:**

The effects of IL-35 on cell proliferation, cytokine expression, and histamine release in a human mast cell line (HMC­1) were investigated by CCK8, ELISA, or RT-PCR. The phosphorylation levels of ERK1/2, p38, and JNK1/2, in PMA plus A23187 induced HMC-1 cells was detected by Western Blot.

**Results:**

We found that IL-35 significantly inhibited the proliferation of HMC-1 cells stimulated by PMA and A23187. IL-35 also down-regulates the release of histamine and the mRNA expression of IL-6 and IL-17 in activated HMC-1. Furthermore, IL-35 markedly inhibited the phosphorylation levels of ERK1/2, p38, and JNK1/2, in PMA plus A23187 induced HMC-1 cells.

**Conclusions:**

This study provides the first observations on the inhibitory and anti-inflammatory effect of IL-35 in activated HMC-1 cells. We suggest that IL35 may play an inhibited role in the pathogenesis of CSU.

## Background

Chronic spontaneous urticarial (CSU), a mast cell-driven disease, is defined as the spontaneous appearance of weals, angioedema or both for > 6 weeks for unknown or known causes [1]. Mast cells, as one of the major effector cells in the immune response system, plays an important role in the pathogenesis of CSU [2]. Although several studies had indicated that activated mast cells could release histamine and other cytokines, such as interleukin-6 (IL-6), tumor necrosis factor (TNF)-α or vascular endothelial growth factor (VEGF), which could be involved in inducing or developing CSU symptoms [3, 4]. However, the actual pathogenesis of CSU is not yet clear.

IL-35 is a newly anti-inflammatory cytokine that belongs to the IL-12 family and is composed of two subunits: Epstein–Barr virus-induced gene 3 (EBI3) and IL-12p35 [5]. Collison et al. have found that IL-35 signals through a unique heterodimer or homodimers receptor of IL-12R-β2 and gp130 or each chain [6]. IL-35 is widely recognized as a definite immune suppressor with a huge potential for suppression. Tregs are the main resources for IL-35 secretion [7]. Abundant evidence had indicated that IL-35 could suppress the activities of Th1 and Th17 cells and plays a crucial role in the pathogenesis of many types of autoimmune diseases, such as inflammatory bowel disease (IBD) [8], rheumatoid arthritis (RA) [9], and systemic lupus erythematosus (SLE) [10]. IL-35 could inhibit experimental colitis [5] and dampen collagen-induced arthritis (CIA) in DBA/1 mice via suppression of Th17 cells [11]. Collison et al. reported that IL-35 primarily inhibits T-cell proliferation and further amplify T-cell effects by inducing a regulatory population capable of suppressing immune responses via IL-35 [12]. However, the effects of IL-35 in the pathogenesis of CSU has not yet been elucidated.

In our previous study, we found the decreased IL-35 serum levels in CSU patients, and the serum IL-35 levels were significantly increased in CSU patients after conventional treatment [13]. Therefore, we suggest that IL-35 may serve as anti-inflammatory cytokines, and play a role in the pathogenesis of CSU. To explore the role of IL-35 in the pathogenic mechanism of CSU, this study investigated the inhibited effects of IL-35 on phorbol 12-myristate 13-acetate (PMA) plus A23187 (calcium ionophore)-stimulated human mast cell line (HMC-1) cells.

## Materials and methods

### Cell culture and stimulation

The HMC-1 cells were purchased from EK-Bioscience of Shanghai company and cultured in IMDM with 100 U/ml of penicillin and streptomycin, and 10% fetal bovine serum (FBS) at 37 °C in 5% CO_2_. The HMC­1 cells were treated with recombinant human IL-35 (1–1000 ng/ml) for 6 h. The cells were then stimulated with 50 nM of PMA plus 1 μM of A23187 and incubated at 37 °C for the indicated periods (15 min-8 h).

### Assay for the receptor of IL-35 (IL-12R-β2 and gp130)

The DNA was isolated from HMC-1 cells using DNA Reagent Kit (Tiangen Biological Manufacture Co. Ltd., Beijing, China). The primers for IL-12R-β2 and gp130 were designed by ourselves. The forward primer sequence for IL-12R-β2 was 5′-AGAGGCGATGTGACTGTGAA-3′. The reverse primer sequence for IL-12R-β2 was 5′-TCAGGGGTGAGGTTGATTCC-3′. The forward primer sequence for gp130 was 5′-CCAGTGGTCACCTCACACTC-3′. The reverse primer sequence for gp130 was 5′-GGGCAAAATACCATCACCGC-3′. The conditions were 94 °C for 90 s, and 94 °C for 30 s and 55 °C for 30 s of 35 cycles with an extension at 72 °C for 5 min.

The total protein samples of HMC-1 cells were extracted. Protein samples of 40 μg were electrophoresed on 6% Tris–glycine gels, subjected to sodium dodecyl sulfate-polyacrylamide gel electrophoresis, and transferred to polyvinylidene fluoride membranes. Subsequently, membranes were incubated with primary antibody (IL-12R-β2 and gp130) at 4 °C overnight and with the appropriate horseradish peroxidase-conjugated secondary antibody for 1 h. The expression of IL-12R-β2 and gp130 was determined with enhanced chemiluminescence reagents and exposure to a Kodak x-ray film (Eastman Kodak, Rochester, New York).

### Cell viability

Cell Counting Kit-8 (CCK8, WST, China) was used for cell viability assay. Firstly, HMC-1 cells were seeded in a 96-wells plate at the density of 103 cells/well in 100 μl medium. Cells were treated with recombinant human IL-35 (1, 10, 100, 1000 ng/ml) for 6 h at the beginning of the test and cell viabilities were determined. In addition, cells were treated with different concentrations of IL-35 (1, 10, 50, 100, 1000 ng/ml) for 6 h before stimulation with 50 nM of PMA plus 1 μM of A23187 and incubated at 37 °C for 8 h. And then 10 μl CCK8 was added to each well and incubated for 1 h, and the optical density (OD) value was determined by a microplate reader at 450 nm.

### Histamine assay

Histamine levels from HMC-1 cells were determined using Human Histamine Elisa Assay Kit (Nanjing Jiancheng Bioengineering Institute, Nanjing, China) following the manufacturer’s instruction. The optical density (OD) was determined at 450 nm in a microplate reader. The histamine release is present as a percentage of total [released/(intracellular + released)] × 100%.

### Real-time quantitative PCR

HMC-1 cells were seeded in a 6-wells plate and then treated with recombinant human IL-35 (50 and 100 ng/ml) for 6 h before stimulation with 50 nM of PMA plus 1 μM of A23187 and incubated at 37 °C for 8 h. The mRNA levels of IL-4, IL-6, IL-17, IFN-γ, and TNF-α in HMC-1 cells from different groups were determined by real-time quantitative PCR. Total RNA was extracted by Trizol reagent (Invitrogen Corp, Carlsbad, CA, USA) according to the manufacturer’s instructions. The cDNA was synthesized from the total RNA by using RT reagent Kit with gDNA Eraser (Takara, Dalian, China). cDNA samples were amplified in a 20 μl reaction volume containing 10 μl of 2× SYBR GreenMaster Mix (Takara, Dalian, China), 2 μl of cDNA and 0.25 μM qPCR primers. The following primers were used: IL-4 (P216616, Bioneer, Inc., Daejeon, Korea); IL-6 (P211161, Bioneer, Inc., Daejeon, Korea); IL-17 (P291322, Bioneer, Inc., Daejeon, Korea); INF-γ (Catalog: HQP009467, GeneCopoeia, USA); TNF-α (P237423, Bioneer, Inc., Daejeon, Korea); GAPDH (5′-CGGAGTCAACGGATTTGGTC-3′ and 5′-CGGTGCCATGGAATTTGCCA-3′). The conditions were 95 °C for 5 min, and 95 °C for 15 s and 60 °C for 30 s of 40 cycles with a final extension at 72 °C for 5 min. The mRNA levels of IL-17 and IL-6were expressed as relative mRNA levels compared with control and determined by the 2^−ΔΔCt^ method.

### Western blot analysis

The expression of mitogen-activated protein kinases (MAPKs) in HMC-1 cells was measured by western blot. HMC-1 cells were treated with recombinant human IL-35(50 and 100 ng/ml) for 6 h before stimulation with 50 nM of PMA plus 1 μM of A23187 and incubated at 37 °C for 15 min. After treatment, cells were collected and lysed in ice-cold RIPA buffer (EMD Millipore, Billerica, Massachusetts) containing 1% phenylmethylsulfonyl fluoride. The samples were vortex mixed for lysis for a few seconds every 15 min at 4 °C for 1 h and centrifuged at 13,000 rpm for 50 min at 4 °C. Then, the samples were heated at 98 °C for 10 min and briefly cooled on ice. Then, the total protein was extracted. Protein samples of 40 μg were electrophoresed on 12% Tris–glycine gels, subjected to sodium dodecyl sulfate-polyacrylamide gel electrophoresis, and transferred to polyvinylidene fluoride membranes. Subsequently, membranes were incubated with primary antibody at 4 °C overnight and with the appropriate horseradish peroxidase-conjugated secondary antibody for 1 h. The expression of MAPKs was determined with enhanced chemiluminescence reagents and exposure to a Kodak x-ray film (Eastman Kodak, Rochester, New York). The results were normalized to the expression of β-actin.

### Statistical analysis

All data were expressed as mean ± SD. One-way analysis of variance, Mann–Whitney U Test or Wilcoxon sign-rank test were used to compare statistical differences between groups. Each experiment was carried out at least 3 times. P < 0.05 was set as statistically significant.

## Results

### IL-35 receptor IL-12R-β2 and gp130 are expressed in HMC-1 cells

**Fig. 1:**
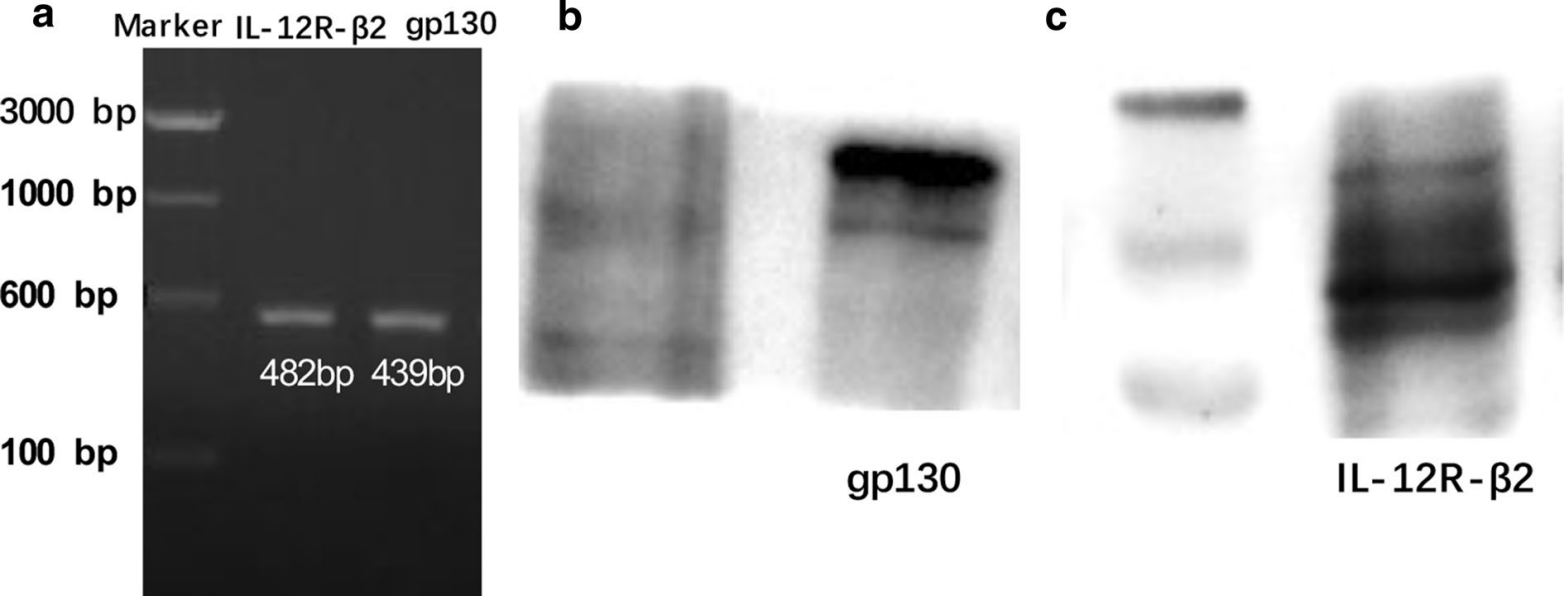
IL-35 receptor IL-12R-β2 and gp130are expressed in HEC-1 cells. **a** The direct expression of IL-35 receptor IL-12R-β2 and gp130 inHMC-1 were detected by PCR. **b**, **c** The expression of IL-35 receptor IL-12R-β2 and gp130 inHMC-1 were detected by western blot

To better establish the relevance of the IL-35 pathway in CSU, we investigated the direct expression of IL-35 receptor IL-12R-β2 and gp130 in HMC-1. By PCR and western blot analysis, we established the expression of IL-12R-β2 and gp130 in HMC-1 (Fig. 1).

### Regulatory effect of IL-35 on the proliferation of HMC-1 cells

**Fig. 2:**
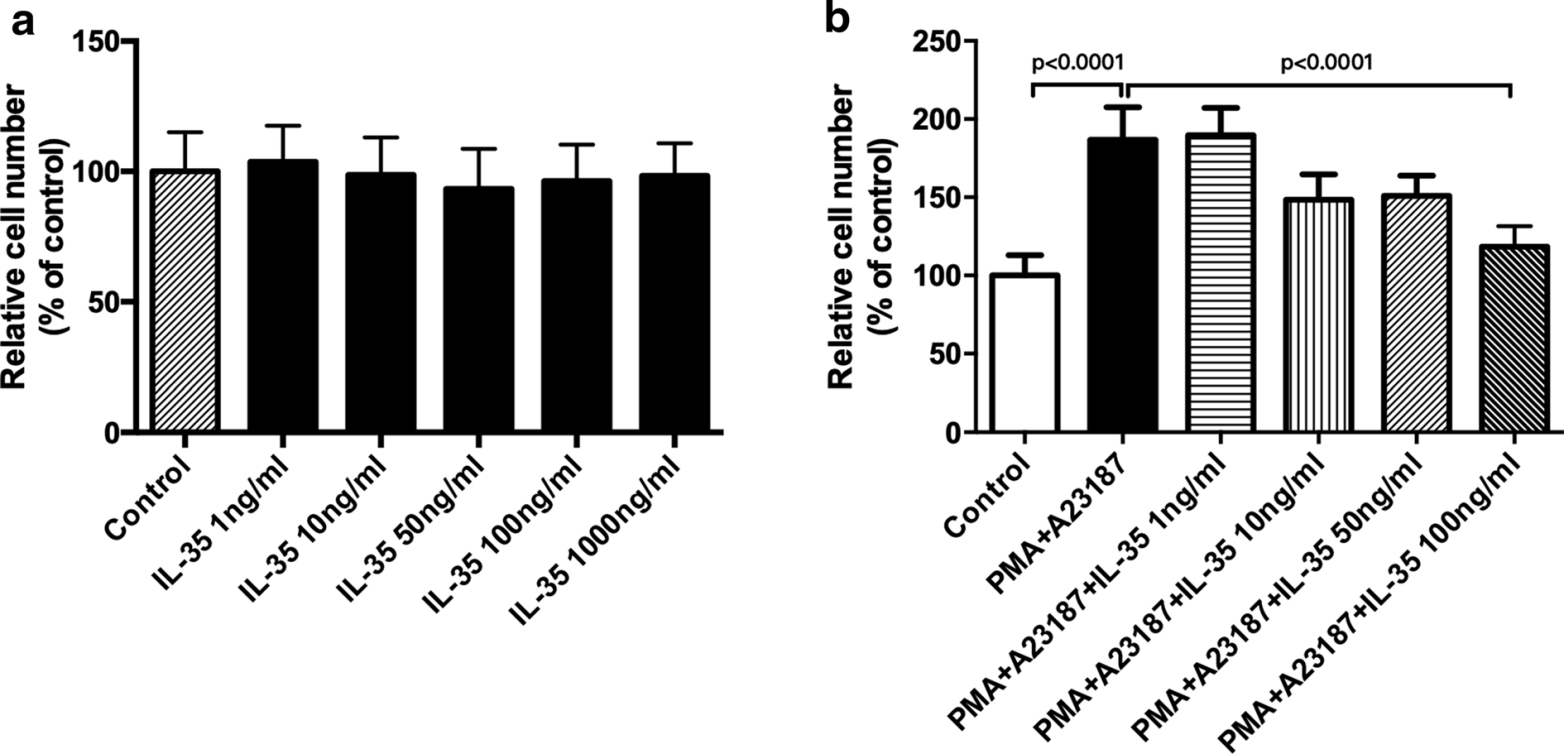
Regulatory effect of IL-35 on the proliferation of HMC-1 cells. **a** HMC-1 cells were treated with IL-35 (1–1000 ng/ml) for 6 h. Cell viability was measured by CCK8. **b** HMC-1 cells were treated with IL-35 (1–100 ng/ml) for 6 h and stimulated with PMA (50 nM) plus A23187 (1 μM) for 8 h. Cell viability was measured by CCK8. All data are expressed as mean ± SD. P values are based on the One-way analysis of variance. n = 10

First of all, the cytotoxicity of IL-35 was evaluated using CCK8 assay, and IL-35 was found not to affect HMC-1 cell viability at concentrations of 1 to 1000 ng/ml (Fig. 2a). After that, we clarified whether IL-35 could regulate mast cell proliferation stimulated by PMA and A23187. As shown in Fig. 2b, IL-35, at a concentration of 100 ng/ml, significantly inhibited the HMC-1 cell viability stimulated by PMA and A23187.

### IL-35 inhibited the histamine releases in HMC-1 stimulated by PMA and A23187

**Fig. 3:**
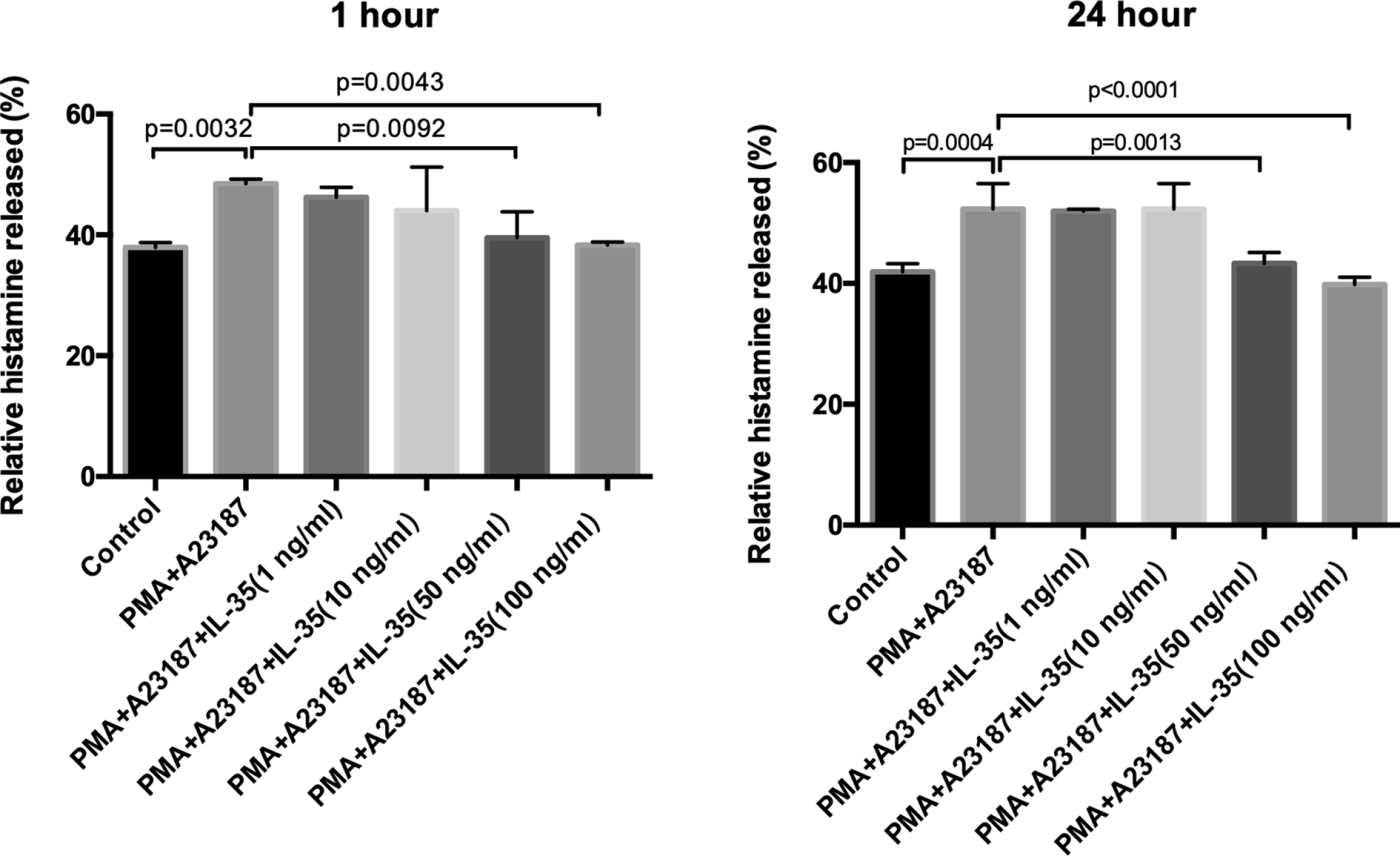
IL-35 inhibited the histamine releases in HMC-1 stimulated by PMA and A23187. HMC-1 cells were treated with recombinant IL-35 (1 ng/ml, 10 ng/ml, 50 ng/ml and 100 ng/ml) for 12 h and stimulated with PMA (50 nM) plus A23187 (1 μM) for1h or 24 h. After that, the histamine was detected by a Histamine assay kit. IL-35 (50 ng/ml and 100 ng/ml) inhibited the histamine releases in HMC-1 cells. All data are expressed as mean ± SD. P values are based on the One-way analysis of variance. n = 6

To explore the role of IL-35 in the HMC-1, we detected the release of histamine in HMC-1 stimulated by PMA and A23187. As presented in Fig. 3, IL-35 significantly inhibited the histamine releases in HMC-1 stimulated by PMA and A23187 when compared with PMA plus A23187 controls.

### IL-35 down-regulates the mRNA expression of IL-6 and IL-17 in HMC-1 stimulated by PMA and A23187

**Fig. 4:**
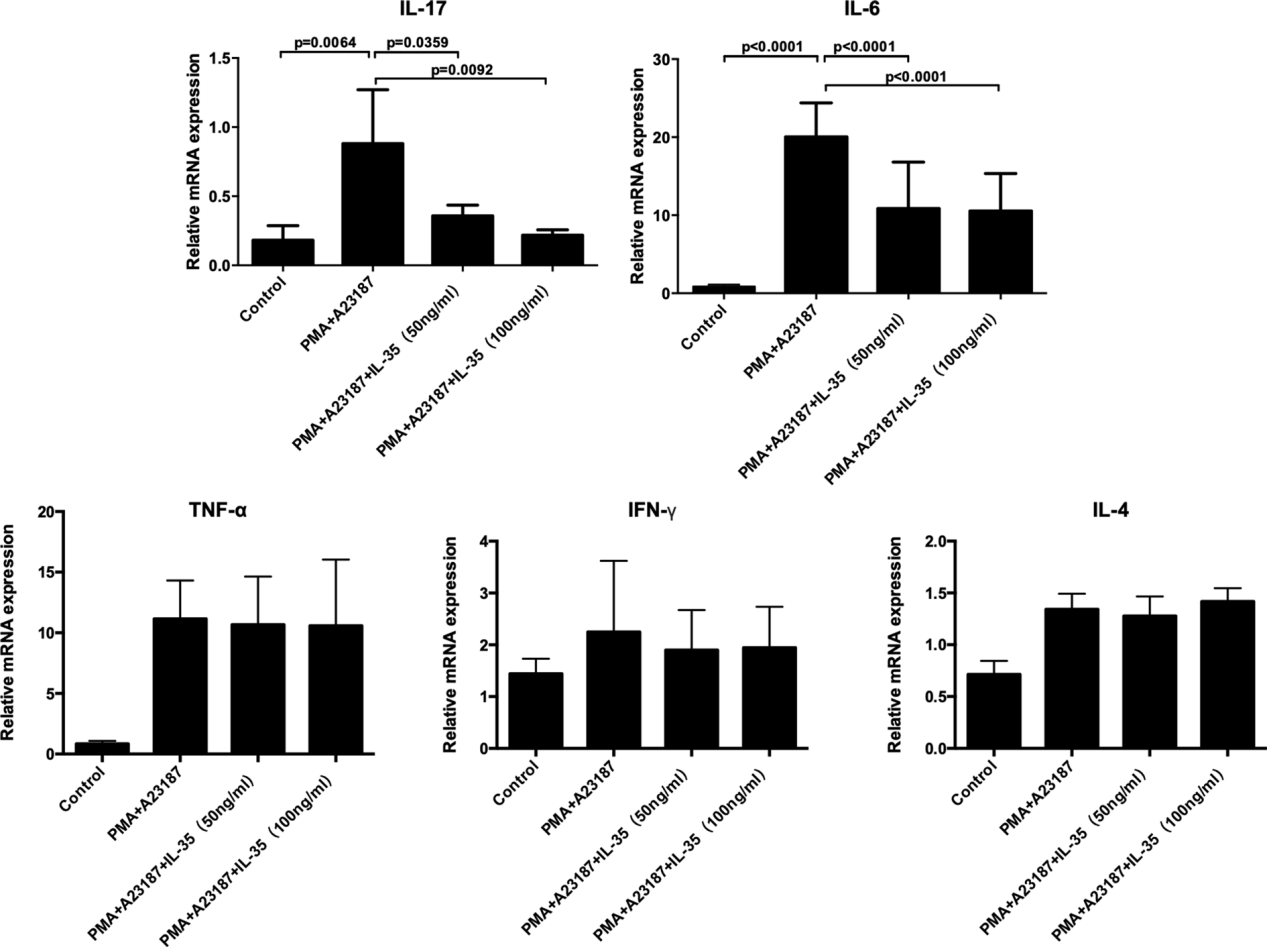
IL-35 down-regulates the mRNA expression of IL-6 and IL-17 in HMC-1 stimulated by PMA and A23187. HMC-1 cells were treated with recombinant IL-35 (50 ng/ml and 100 ng/ml) for 6 h and stimulated with PMA (50 nM) plus A23187 (1 μM) for 8 h. After that, mRNA expression of IL-4, IL-6, IL-17, IFN-γ, and TNF-α in HMC-1 cells was measured by Real-time quantitative PCR. IL-35 inhibited the expression of IL-6 and IL-17 mRNA levels in HMC-1 cells. The mRNA expression of IL-4, IFN-γ, and TNF-α in simulated HMC-1 could not be inhibited by IL-35. All data are expressed as mean ± SD. P values are based on the One-way analysis of variance. n = 10

To explore the role of IL-35 in the HMC-1, we also assayed cytokines mRNA expression (including IL-4, IL-6, IL-17, IFN-γ, and TNF-α) in HMC-1 stimulated by PMA and A23187. As presented in Fig. 4, IL-35 caused a markedly decrease in the mRNA levels of IL-6 and IL-17 in HMC-1 stimulated by PMA and A23187 when compared with controls. However, the mRNA expression of IL-4, IFN-γ, and TNF-α in simulated HMC-1 showed no difference between the groups with or without IL-35 treatment.

### Effects of IL-35 on activation of MAPKs

**Fig. 5:**
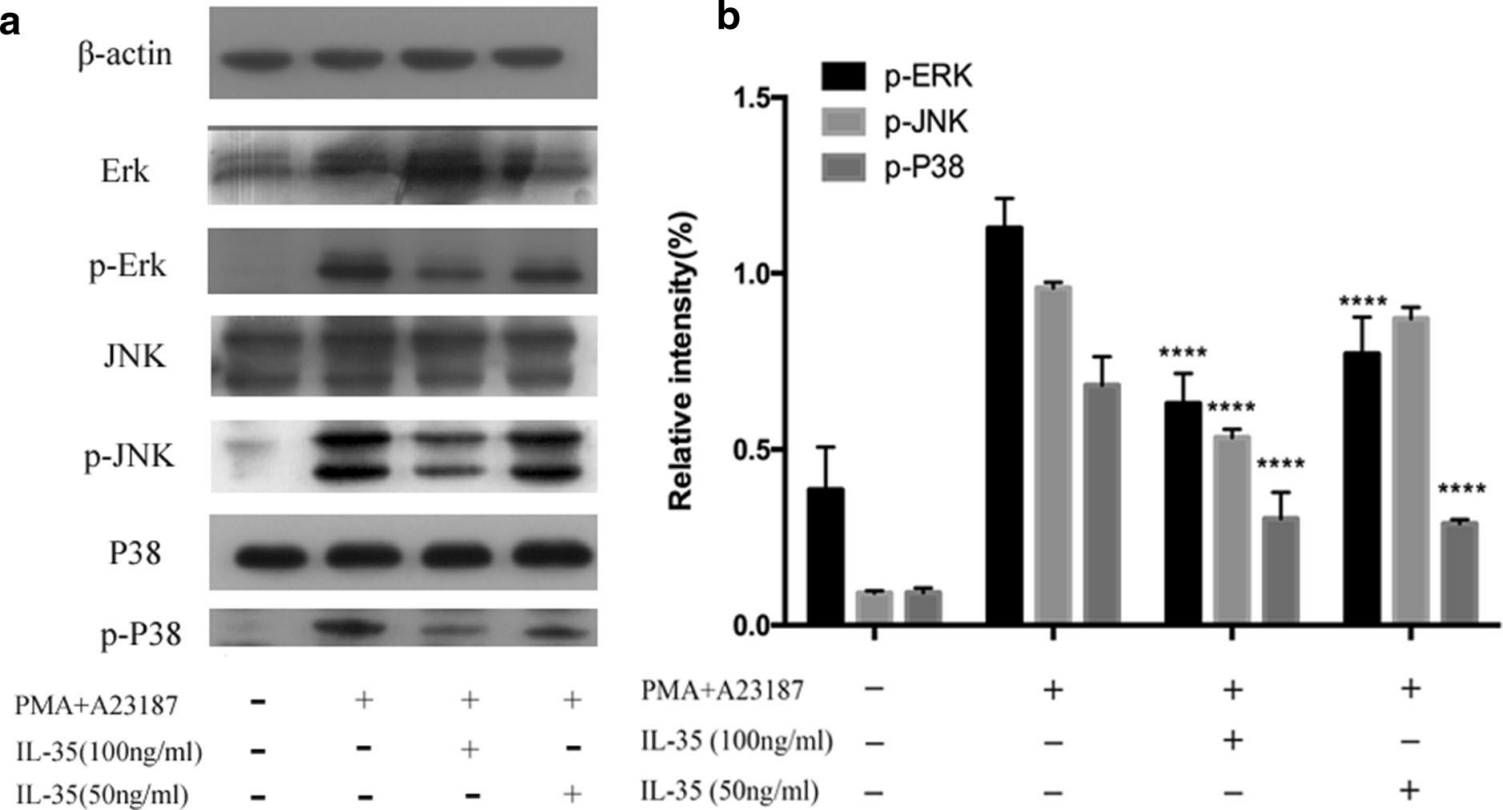
Effect of IL-35 on PMA plus A23187-stimulated MAPKs activation. **a** After pretreatment of IL-35 for 6 h, HMC-1 cells were stimulated by PMA (50 nM) plus A23187 (1 μM) for 15 min for MAPKs activation. Phosphorylation of ERK1/2, JNK1/2, and p38 MAPKs was analyzed by western blotting. **b** The intensity was measured by densitometry. All data are expressed as mean ± SD. P values are based on the One-way analysis of variance. n = 10. ****P < 0.0001 compared with PMA plus A23187 group

To evaluate the mechanisms underlying the effects of IL-35, we examined the potential effects of IL-35 on the activation of MAPKs. The phosphorylation levels of p38, JNK, and ERK were increased after HMC-1 cells treated with PMA plus A23187. As shown in Fig. 5, IL-35 attenuated PMA plus A23187-induced phosphorylation levels of all three types of MAPKs.

## Discussion

IL-35, a novel anti-inflammatory cytokine, is composed of two subunits: EBI3 and IL-12p35 [5]. Previous studies have shown that IL-35 could perform anti-inflammatory and immunosuppressive actions in many autoimmunity diseases and models. Ouyang et al. have found that serum levels of IL-35 and the percentage of CD4+EBI3+ T cells were markedly decreased in patients with active SLE compared with patients with inactive SLE and healthy controls [10]. Niedbala et al. reported that IL-35 can inhibit the development of RA through the suppression of Th17 cells and expansion of Treg cells [11]. IL-35 has also been evaluated the activity of anti-inflammatory in an inflammatory bowel diseases model by reducing colonic gene expression of pro-inflammatory cytokines and Th1/Th17-associated transcription factors [5, 12, 14]. Our previous study also reported that the serum IL-35 levels were significantly decreased in patients with CSU compared with healthy controls and CSU patients after conventional treatment, and indicated that the reduction of IL-35 levels may play an important role in the pathogenesis of CSU [13].

Mast cells, as one of the major effector cells in the immune response system, are the major primary effector cell type in diseases of immediate hypersensitivity [15], including urticarial and angioedema. Mast cells are activated during the immediate IgE-mediated allergic response [16]. Some pro-inflammatory cytokine, such as TNF-α, IL-6, and IL-8 could be derived by activated mast cells and had critical biological roles in the pathogenesis of CSU [4]. Inflammatory cytokines are important factors in chronic inflammation, urticarial, allergy, asthma, and autoimmune diseases. Mast cells play an integral role in the inflammatory response by accumulating at sites of inflammation and mediating the production of inflammatory cytokines, such as IL-6 and IL-8 [17]. In this study, the inhibitory effect of IL-35 on the HMC-1 proliferation is determined by CCK8. The results showed that IL-35 could not affect HMC-1 cell viability at different concentrations. However, IL-35, at a concentration of 100 ng/ml, significantly inhibited the HMC-1 cell proliferation induced by PMA and A23187. Activated mast cells release inflammatory mediators including tryptase, histamine, heparin, leukotrienes, and prostaglandins. Mast cells also express the pro-inflammatory cytokine of IL-4, IL-5, IL-6, IL-8, IL-13, and TNF-α [18]. Previous studies have indicated that the reduction of pro-inflammatory cytokine from mast cells is one of the key indicators of reduced inflammatory symptoms [19]. The present study also examined the inhibitory effect of IL-35 on the production of IL-4, IL-6, IL-17, IFN-γ, and TNF-α in HMC-1 cells activated by PMA plus A23187, as these cytokines have powerful inflammatory effects and are released by activated mast cells. As shown in Figs. 3 and 4, IL-35 inhibited the release of histamine and the gene expression of inflammatory cytokines, IL-6 and IL-17 production in HMC-1 cells stimulated with PMA plus A23187 by RT-PCR analysis. Moreover, the mRNA expression of IL-4, IFN-γ, and TNF-α in simulated HMC-1 could not be inhibited by IL-35. Therefore, these data implied that IL-35 exerts markedly inhibited the proliferation and the anti­inflammatory effects in PMA plus A23187-stimulated HMC-1.

MAPKs belong to a family of proline-directed serine/threonine protein kinases that play an important signaling pathway in immune responses [20]. Three major factors, ERKs, p38, and stress-activated protein kinases (SAPKs)/JNK [17], that mediate the MAPK pathways have been identified in mammals. Although the precise signaling pathways among ERK, JNK, and p38 are still unclear. It has been reported that ERK, p38, and JNK are activated in response to extracellular stimuli and perform different functions, including mediation of apoptosis, proliferation, and inflammation [21]. To explore the mechanism involved in the inhibitory and anti-inflammatory effect of IL-35in HMC-1, we investigated the activation of three members (ERK, p38, and JNK) of MAPKs on PMA plus A23187-stimulated HMC-1 cells. Results of the current study demonstrated that the activities of three members (ERK, p38, and JNK) of MAPKs were increased by PMA plus A23187, and that IL-35 significantly inhibited the phosphorylation levels of ERK1/2, p38, and JNK1/2, in PMA and A23187 induced HMC-1 cells. All the above data suggested that IL-35 inhibited the proliferation and exerted anti­inflammatory effects by decreases IL-6 and IL-17 production in PMA plus A23187-stimulated HMC-1 via the inhibition of ERK1/2, p38, and JNK1/2 activation.

To summarize our results, this study provides the first observations on the inhibitory role of IL-35 in HMC-1 cells. IL-35 significantly inhibited the proliferation and the production of histamine, IL-6, and IL-17 in PMA plus A23187-stimulated HMC-1 cells. Furthermore, IL-35 inhibited the ERK1/2, p38, and JNK1/2 pathways. Therefore, we suggest that IL-35 may serve as anti-inflammatory cytokines, and play a role in the pathogenesis of CSU.

## Data Availability

All data generated or analyzed during this study are included in this published article.

## Abbreviations

CSU: Chronic spontaneous urticarial
IL: Interleukin
EBI3: Epstein–Barr virus-induced gene 3
IBD: Inflammatory bowel disease
RA: Rheumatoid arthritis
SLE: Systemic lupus erythematosus
CIA: Collagen-induced arthritis
AD: Atopic dermatitis
HMC-1: Human mast cell line
PMA: Phorbol 12-myristate 13-acetate
ELISA: Enzyme-linked immunosorbent assay
MAPKs: Mitogen-activated protein kinases
